# Shining a light on the road towards conducting principle-based co-production research in rehabilitation

**DOI:** 10.3389/fresc.2024.1386746

**Published:** 2024-04-10

**Authors:** John A. Bourke, Peter Bragge, Jo River, K. Anne Sinnott Jerram, Mohit Arora, James W. Middleton

**Affiliations:** ^1^ John Walsh Centre for Rehabilitation Research, Northern Sydney Local Health District, St Leonards, NSW, Australia; ^2^ Faculty of Medicine and Health, The Kolling Institute, The University of Sydney, Sydney, NSW, Australia; ^3^ Monash Sustainable Development Institute, Monash University, Melbourne, VIC, Australia; ^4^ Faculty of Health, University of Technology Sydney, Sydney, NSW, Australia; ^5^ Northern Sydney Local Health District, Macquarie Hospital, Sydney, NSW, Australia; ^6^ Royal Rehab, Ryde, NSW, Australia; ^7^ State Spinal Cord Injury Service, NSW Agency for Clinical Innovation, St Leonards, NSW, Australia

**Keywords:** co-production research, lived experience research, research partnership(s), epistemic justice, capacity building, rehabilitation research design

## Abstract

Moving from participatory approaches incorporating co-design to co-production in health research involves a commitment to full engagement and partnership with people with lived experience through all stages of the research process—start to finish. However, despite the increased enthusiasm and proliferation of research that involves co-production, practice remains challenging, due in part to the lack of consensus on what constitutes co-production, a lack of guidance about the practical steps of applying this approach in respect to diverse research methods from multiple paradigms, and structural barriers within academia research landscape. To navigate the challenges in conducting co-produced research, it has been recommended that attention be paid to focusing and operationalising the underpinning principles and aspirations of co-production research, to aid translation into practice. In this article, we describe some fundamental principles essential to conducting co-production research (sharing power, relational resilience, and adopting a learning mindset) and provide tangible, practical strategies, and processes to engage these values. In doing so, we hope to support rehabilitation researchers who wish to engage in co-production to foster a more equitable, ethical, and impactful collaboration with people with lived experience and those involved in their circle of care.

## 1 Introduction

The idea of participatory research, where people with lived experience and rehabilitation researchers partner together in the planning, design, conduct, dissemination and implementation of research, has attracted increasing attention and enthusiasm in recent decades (1–3). The push for participation, in part, is due to increasing recognition that partnering with people with lived experience increases the relevance of research priorities and outcomes (4, 5) and raises the quality of interpretation and knowledge translation (6) as it is more reflective of lived reality and can bridge the gap between research and practice (4, 7, 8).

There are many participatory approaches with concepts and terms often used interchangeably (9, 10). This has led to what Williams et al. (2) termed *cobliquity*, which refers to the emergence of “a plethora of “co” words, promoting a conflation of meanings and practices from different collaborative traditions” (p. 2). Indeed, each participatory approach has a distinct history, concepts, and commitments to power-sharing with people with lived experience (11–13). Two approaches that are commonly used are co-design and co-production. While there is no consensus regarding use of these terms (14), co-design often refers to collaborative approaches with design elements, and has its origins in Scandinavian “co-operative” or “participatory” design with end users of products, services, and workplaces (15, 16). Co-design involves partnership with people with lived experience in one or more stages of the research process (17)—although a substantive approach may encompass all stages (15). The increasing popularity of co-design no doubt reflects a renewed focus on person-centred and collaborative models of healthcare provision and a greater involvement of patients and community members in health research (18). Further, changes in the research management landscape (such as requirements of ethics committees and health research funding bodies) mean that there is now a greater focus on partnerships (8).

Co-production, on the other hand, which originated in US social care and justice movements (19) refers to a collaborative approach that centres reflective dialogue (15, 20). There are growing demands from consumer and disability movements for co-production research. Co-production has often been described as the “gold standard” for participatory research (21), and motivated by an “egalitarian imperative” (2). Co-production has strong commitments to collaboration and power-sharing throughout all stages of the research process (20), and can also involve development of research agendas with affected communities (15), establishing health policy (22) and translating evidence into action (11). The push for co-production research has largely come out of recognition of human rights violations against people with lived experience (23, 24) and a central desire to redistribute power in the social relations of research to promote epistemic and health justice (25–27).

Despite the increasing attention on, and proliferation of co-production research, practice remains challenging (28), which is in part due to the lack of consensus on what constitutes co-production in the context of health research (2, 9). Challenges include a lack of guidance in relation to the practical steps of applying this approach across diverse research methods from multiple paradigms (1, 8), and structural barriers within academia and funding landscapes, which are often conflicting with the practice of co-production research (27, 28). To navigate the challenges in conducting co-produced research, it has been recommended that attention be paid to advocating for and operationalising the values, principles, and aspirations of co-production to aid translation into practice with lived experience communities—not as a linear approach but rather as an ethos that shapes practice (2, 9, 28). As such, co-production has been described as a principles-based approach, and a process which can draw on multiple methods from multiple paradigms (1, 8, 26).

To this end, numerous efforts have been made to articulate and classify the underlying principles and values conducive to co-production research [for example, see Hickey et al. (29) and Gainforth et al. (4)]. Notable commonalties amongst these principles include the redistribution and sharing of power—where the research is jointly owned and people work together to achieve co-determined outcomes (4, 29, 30). This is accompanied by relationship building and maintenance to enable contribution with power sharing (1, 3, 27). The adoption of a learning mindset—whereby team members embrace different perspectives and build capability through undertaking an iterative approach and being open to adjustments based on ongoing reciprocal feedback, is also important (28, 31, 32).

Although this broad conceptualisation of practice can help guide researchers, they might nonetheless be experienced as rather abstract and hard to operationalise in practice. While some existing resources suggest recommendations on how to conduct co-production research [for example, see Hickey et al. (29), McKercher (33) and Bellingham (15)], there remains a need for guidance regarding how researchers might navigate the everyday challenges of co-production research. In the following section, we, as a group of researchers engaged in co-production research from lived experience and “conventional” (non-lived experience) positions, present some common challenges to conducting co-production research and some pragmatic strategies we have used to address these (See Table 1).

**Table 1:** Overview of principles, challenges, strategies, and recommended readings.

Principles	Challenges	Strategies	Recommended reading
1. Navigating power dynamics	• Power and positionalities shape knowledge creation and knowledge value. • People with lived experience have historically been excluded and invalidated in research.	• Be deliberately reflective and attentive to status throughout the research lifecycle. • Have honest, high-quality conversations between those with lived experience and researchers. • Establishing a democratic governance framework. • Building flexibility into timelines, support those with lived experienced to take the lead on projects or project components, set clear meeting agendas. • Include adequate representation and remuneration of people with lived experience. • Building research skillsets and/or developing research career pathways for those with lived experience.	• Plamondon et al. (30) • Staniszewska et al. (34) • Bell and Pahl (3) • Flinders et al. (7) • Bourke et al. (35) • Williams et al. (2)
2. Building relational resilience	• Democratic rationales for co-production research require conventional researchers to employ more equal relations than they may have been accustomed to. • Traditional academic and research funding practice can lack the flexibility required to build and maintain meaningful relationships.	• Invest time with community partners even before initiating the research. • Adopting an approach of “generous hospitality” to ensures that people with lived experience feel welcome. • Identify joint priorities, as well as support dialogue around appropriate and acceptable research questions, methodologies, timelines, roles, governance structures, and dissemination strategies. • Accessible and appropriate ways for people to digest and contribute information. • Ensure venues, information mediums and communication and timeframes are accessible.	• River et al. (27) • McKercher (33) • Cooke et al. (36) • Bellingham et al. (15) • Daya et al. (37) • Happell et al. (21) • Middleton et al. (17)
3. Adopting a learning mindset	• Real world practicalities (e.g., complex power relations, competing or conflicting intentions, lack of organisational support, and expectations and priorities which can frustrate the research process). • Unexpected barriers and challenges (e.g., contacting those lived experience, navigating the sharing of decision making, experiencing instances of not knowing what to do next).	• Learn through doing—be as flexible and adaptable as possible. • Embrace and practice dialogue (act of listening, sharing, and acknowledging others point of view) and iteration (being open to adjustments based on ongoing reciprocal feedback, and seeing and valuing different perspectives).Make time to reflect on how the group is working and whether initial ideas about communication and relationships are being maintained and/or need development.	• Langley et al. (11, 31, 32) • Hickey et al. (29) • McKercher (33) • Gainforth et al. (4) • Hoekstra et al. (5) • Sibley et al. (38)

### 1.1 Navigating power dynamics

#### 1.1.1 Challenge

Breaking down power imbalance and structural inequities for power sharing.

Plamondon et al. (30) argue that power is the overarching and essential problem of research co-production. Not only is the research process (involving the systematic nature of knowledge enquiry) founded on human relationships, “power and positionalities shape who and what is seen, privileged, and legitimized as worthy of research and implementation attention and resources” (p. 37). People with lived experience have historically been excluded and invalidated in research (28, 34). The influence of research context also matters (2), and traditionally, power has resided with conventional researchers due to structural inequalities and embedded hierarchies within research institutions and structures, such as existing in universities and research funding systems, which often reflect society more broadly and structural inequalities (28). However, reflecting on power relationships is becoming more common and receiving more attention in the literature, and there are now several ways to acknowledge and work towards mitigating power inequities and practicing more equitable co-production research (2, 34).

#### 1.1.2 Strategies

There are a variety of ways in which researchers can promote parity in co-production teams. At a foundational level, conventional researchers (as the traditional power holders) need to be deliberately reflective and attentive to their status throughout the research lifecycle and lived experience perspectives need to be elevated. As McKercher (33) states “elevating the voices and contributions of people with lived experience means challenging power differences. Including what is considered evidence, who gets heard, who gets to decide, and who is in the room”. Staniszewska et al. (34) argues that honest, high quality conversations between those with lived experience and researchers that take account of how power works in research can serve to create a more fertile and kinder context for co-production research. At a practical level, efforts to address power inequities may include establishing an open governance framework, that clearly articulates the decision-making process and how disagreements will be navigated. It also includes building flexibility into timelines, supporting those with lived experienced to take the lead on projects or project components, setting clear meeting agendas and deciding on what time should be spent on particular topics/activities (36). Two further important equity efforts are to include adequate numbers of people with lived experience and remuneration for lived experience (39), and ensuring that co-production projects build skillsets and capacity in all members of the team (e.g., all members are trained in co-production research principles and practices), which may take a longer commitment beyond the life of the project (35, 40).

### 1.2 Building relational resilience

#### 1.2.1 Challenge

Being able to invest the time, effort and resources necessary to build trust and understanding of the needs of people with the lived experience.

Rycroft-Malone et al. (1) suggest authentic co-production requires a “sustained investment in building and maintaining meaningful relationships” (p. 291). As such, the building of trust and a shared vision for a research project with people with the lived experience requires more time, effort and resources compared to conventional research methods (27). This investment in building meaningful relationships can be challenging, with democratic rationales for co-production research requiring conventional researchers to employ more equal relations than they may have been accustomed to (2). In addition, traditional academic and research funding practice can lack the flexibility required to build and maintain meaningful relationships (4), which may make it difficult to estimate in funding requests or lead to costs stretching beyond a proposed budget, and may also be at odds with the tight timeframes imposed by funders and universities (4, 27). However, while challenging, investing time, energy and resources in building relationships and understanding motivations and intentions for the project, is nonetheless necessary for establishing authentic partnerships (27), and for co-production research to ultimately flourish or fail (1).

#### 1.2.2 Strategies

Building trusting relationships is a key strategy of co-production teams. River et al. (27) found that relational connection in research teams is essential to enabling sharing of lived expertise, which is not only conceptual, but also as Bell and Pahl (3) have argued, tacit, embodied, personal and emotional. Building relationships often requires researchers to invest time with community partners even before initiating the research. When preparing, taking the opportunity to discuss with affected communities who should be involved and what the focus of the research might be is useful for ensuring research relevance and supporting later implementation efforts (15). Once established, taking time and paying attention to relationships can also support “relational resilience” to navigate conflict and team disagreement when and if it arises (27). McKercher (33) suggests adopting an approach of “generous hospitality” to ensures that people with lived experience feel welcome and that their hopefulness for equity and change is “cared for”. McKercher (33) notes that basics are vital, including ensuring that we know people's names, offering food and drinks, and welcoming everyone each time. Cooke et al. (36) also emphasises that in relationships, researchers be nimble, honest, and reciprocal—endeavouring to listen and offer perspectives to the dialogue.

Through building relationships, conventional researchers, who are motivated by values of equity, can begin to develop an understanding of the motivation of partners with lived experience (a process inevitably related to power, see below). Bellingham et al. (15) also note that conventional researchers may hold “unexamined and unarticulated intentions that are at odds with the intentions of lived experience researchers”. While conventional researchers may see lived experience input as an “add-on”, lived experience researchers may view it as promoting epistemic justice, which is central to equity and social change (41). Early articulations of intentions and motivations for a project can help co-production teams to identify joint priorities, as well as support dialogue around appropriate and acceptable research questions, methodologies, timelines, roles, governance structures, and dissemination strategies (15). In these discussions, teams must not only value diverse forms of expertise, but also welcome divergent views as disruptive, and potentially tense dialogue, which is indeed a strength of co-production as it encourages innovation and helps to ensure relevance and resonance to lived experience communities (37, 42). Having a process for resolving disputes at the outset can potentially help enable and depersonalise disruptive and tense dialogue.

Facilitating effective communication is essential and requires several pragmatic considerations. For example, thinking carefully about how to optimally share, use, and capture information (32), offering appropriate ways and mediums for people to digest and contribute. Giving consideration to the meeting location (15) is important for making engagement opportunities accessible and inclusive for those of the lived experience to participate fully. Further considerations (in a rehabilitation context) include how various biopsychosocial consequences of impairments might impact on the associated environmental factors and practice of relationship building, timeframes and accessibility (4). Building relationships can be of value not just for one research project but for research efforts over time. Furthermore, building relationships can be critical in brokering greater participant recruitment, building research skillsets and/or developing research career pathways for those with lived experience (5, 35).

### 1.3 Adopting a learning mindset

#### 1.3.1 Challenge

Co-production research ideals are often challenged by real world practicalities.

The transformative promise of authentic co-production research (in the research space, and through the renewal of wider scientific democracy) is often stymied by complex power relations, competing or conflicting intentions, lack of organisational support, and expectations and priorities, which can frustrate the research process (7). Many researchers engaging in co-production acknowledge such difficulties and admit that conducting “perfect” co-production research is perhaps a quixotic quest (28).

#### 1.3.2 Strategies

Adopting a learning mindset is a key consideration (33). As Langley et al. (32) suggest, “co-production research is not a technique you apply rightly or wrongly, but a journey of learning, and it is not a journey you make alone” (p. 112). So much about co-production can be learnt through doing. Because co-production does not have a prescribed method nor a checklist, research can employ a flexible and adaptable approach. Practical experience can be gleaned through starting what is possible for often limited resources and expertise (15). When doing so, Langley et al. (32) suggest that two important processes intractably linked to the core values of co-production research include dialogue and iteration. Dialogue refers to the interaction and act of listening, sharing, and acknowledging others point of view. This includes acknowledgement and engagement with lived expertise of lived experience researchers, as well as acknowledgement and engagement with “learned” expertise of conventional researchers. Three qualities which can greatly benefit listening and acknowledgement of diverse perspectives include *flexibility, adaptability, and humility*. Iteration refers to the applied use of such interaction and incorporation of feedback with ongoing learning, which results from step change.

Embracing an iterative approach, being open to adjustments based on ongoing reciprocal feedback, and seeing and valuing different perspectives is essential (5, 38). For example, when reflecting on their experiences of bringing co-production principles into practice, Farr et al. (28) recommend that making time to reflect on how the group is working and whether initial ideas about communication and relationships are being maintained and/or need development is vital to ensuring that people are being heard and their needs are addressed. Furthermore, seeing and valuing different perspectives goes both ways between those with lived experience and conventional researchers (5). People with lived experience might need to be adaptable. This can be a potentially awkward part of the co-production process but very important to realise the two-way nature of the co-production research (38).

## 2 Discussion

With increasing calls for epistemic and health justice, the practicing of authentic, sustainable co-production which focuses on equality and reciprocity, will likely become the “new normal” and commonplace in health research (10, 43). However, as many researchers have reported, co-production research can be challenging in practice (15, 22, 34). In order to avoid tokenism, and conduct of co-production “in name if not always in deed” (1) (p. 290), attention to the values and principles of co-production research is required, but also strategies to manage common challenges related to power dynamics, relationships, and real-world contexts.

It must also be acknowledged that the practice of co-production research takes place within a vast research ecosystem, which does not traditionally facilitate the egalitarian nature of co-produced research (1, 2). The practice of co-production research is often portrayed as being inherently more difficult, time consuming and resource intensive than conventional research [for example, see Oliver (42)]. However, many now argue that such risks and challenges of co-production research are not the result of “bad practice” *per se* (2), but instead result from systematic barriers within the research landscape. Common barriers may include the embedded hierarchies and structural inequalities in universities, culture of public service institutions and research funding systems, inflexible funding timelines, and valuing of non-typical research outputs and metrics) (2, 7, 28, 44).

However, there is a great desire to transform the practice, culture, and structures of the research ecosystem to be more encompassing of authentic co-production research (28, 34). Increasing discussion is challenging multiple areas across the research ecosystem, including how funders can be more enabling of co-production research (1), how the public, researchers and policymakers can work better together to co-produce and implement evidence-based policy (22), and how co-produced models of research commissioning within public health can improve the setting of research agendas (44). Furthermore, there is a current lack of practical co-production research evaluation frameworks. To address this gap there are increasing and concerted efforts being made to develop, test, and refine evaluation frameworks. For example, the Research Quality Plus for Co-Production (RQ + 4 Co-Pro) Framework (45), and for co-design, the Preferred Components for Co-design in Research (PRECISE) guideline (46). Having such approaches which aim to help evaluating the quality of participatory research can help both co-producers learn and improve their practice, and provide a greater methodological impetus for co-production research projects to be more widely accepted by funders (47).

While our discussion has mainly focused on co-production research practice, there is growing consensus that co-produced research is a critical mechanism to improve research translation and benefit clinical practice (4, 48, 49). To date, participatory research within rehabilitation settings has reported successful achievement of focused clinical-level outcomes, such as having lived experience involvement in clinical service and technology development (50, 51). The challenge now for the rehabilitation research community is to engage in genuine epistemic, or knowledge, justice—founded on (but not limited to) the principles of power sharing, relationship building and adaptability (2, 4, 52). Epistemic justice is vital and necessary in rehabilitation research, not only to improve the translation of lived experience knowledge to practice, but because partnership with people with lived experience of disability in the production of knowledge actively commits to the rights of disabled people (53), and makes real the demand for “nothing about us without us” in the production of rehabilitation research (4, 54).

## 3 Conclusion

Creating more optimal conditions for co-production research will inevitably require a more equitable approach to research, which challenges our current systems of research production. The multitude of contexts and stakeholders involved throughout the entire research ecosystem will inevitably require creative approaches to co-production research (31). Part of this journey requires researchers to understand some fundamental principles essential to conducting co-production research, and have access to tangible, practical strategies, and processes to properly engage these principles. In this paper, we have offered a few entry-point strategies to support researchers with these common challenges, including strategies to navigate power dynamics via intentional dialogue and clarity for decision-making processes; strategies to build relational resilience in research teams via attention to relationships, intentions, and motivations; and moving beyond perfection to adopting a learning mindset. While by no means an exhaustive list, we hope that rehabilitation researchers who wish to practice co-production research consider these strategies, which aim to foster a more equitable, ethical, and impactful collaboration with rehabilitation communities.

## Data Availability

The original contributions presented in the study are included in the article/Supplementary Material, further inquiries can be directed to the corresponding author.
